# (2,2′-Biquinoline-κ^2^ *N*,*N*′)dichlorido­iron(II)

**DOI:** 10.1107/S1600536809041439

**Published:** 2009-10-17

**Authors:** Narjes Rahimi, Nasser Safari, Vahid Amani, Hamid Reza Khavasi

**Affiliations:** a Department of Chemistry, Shahid Beheshti University, G. C., Evin, Tehran 1983963113, Iran

## Abstract

In the title compound, [FeCl_2_(C_18_H_12_N_2_)], the Fe^II^ atom is four-coordinated in a distorted tetra­hedral arrangement by an *N*,*N*′-bidentate 2,2′-biquinoline ligand and two chloride ions. In the crystal, there are extensive π–π contacts between the pyridine rings [centroid–centroid distances = 3.7611 (3), 3.7603 (4), 3.5292 (4), 3.5336 (5) and 3.6656 (4) Å].

## Related literature

For related structures, see: Amani *et al.* (2009 ▶); Amani, Safari & Khavasi (2007 ▶); Amani, Safari, Khavasi & Mirzaei (2007 ▶); Chan & Baird (2004 ▶); Gibson *et al.* (2002 ▶); Handley *et al.* (2001 ▶); Khavasi *et al.* (2007 ▶, 2008 ▶). For bond-length data, see: Figgis *et al.* (1983 ▶); Kulkarni *et al.* (1998 ▶).
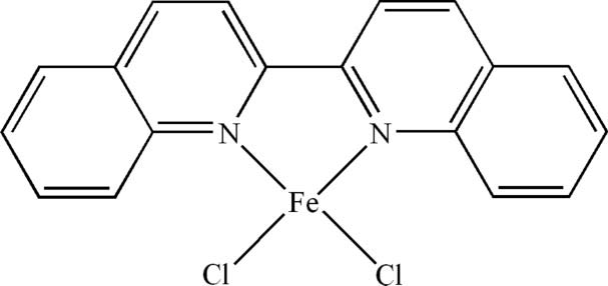


## Experimental

### 

#### Crystal data

[FeCl_2_(C_18_H_12_N_2_)]
*M* *_r_* = 383.05
Monoclinic, 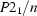

*a* = 7.9777 (6) Å
*b* = 12.2268 (11) Å
*c* = 16.9904 (12) Å
β = 102.899 (6)°
*V* = 1615.5 (2) Å^3^
*Z* = 4
Mo *K*α radiation
μ = 1.26 mm^−1^
*T* = 298 K
0.45 × 0.43 × 0.31 mm

#### Data collection

Stoe IPDS II diffractometer
Absorption correction: numerical (*X-SHAPE*; Stoe & Cie, 2005 ▶) *T*_min_ = 0.577, *T*_max_ = 0.681
13058 measured reflections
4323 independent reflections
3739 reflections with *I* > 2σ(*I*)
*R*_int_ = 0.024

#### Refinement

*R*[*F* ^2^ > 2σ(*F* ^2^)] = 0.037
*wR*(*F* ^2^) = 0.093
*S* = 1.06
4323 reflections
208 parameters
H-atom parameters constrained
Δρ_max_ = 0.52 e Å^−3^
Δρ_min_ = −0.44 e Å^−3^

### 

Data collection: *X-AREA* (Stoe & Cie, 2005 ▶); cell refinement: *X-AREA*; data reduction: *X-RED* (Stoe & Cie, 2005 ▶); program(s) used to solve structure: *SHELXS97* (Sheldrick, 2008 ▶); program(s) used to refine structure: *SHELXL97* (Sheldrick, 2008 ▶); molecular graphics: *ORTEP-3* (Farrugia, 1997 ▶); software used to prepare material for publication: *SHELXL97*.

## Supplementary Material

Crystal structure: contains datablocks I, global. DOI: 10.1107/S1600536809041439/hb5133sup1.cif

Structure factors: contains datablocks I. DOI: 10.1107/S1600536809041439/hb5133Isup2.hkl

Additional supplementary materials:  crystallographic information; 3D view; checkCIF report

## Figures and Tables

**Table:** 

Fe1—N1	2.1051 (14)
Fe1—N2	2.1008 (15)
Fe1—Cl2	2.2265 (6)
Fe1—Cl1	2.2341 (7)

**Table:** 

N2—Fe1—N1	78.06 (6)

**Fig. 1.**
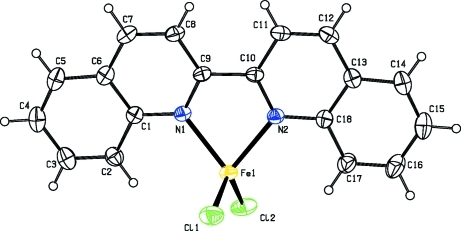
The molecular structure of (I) with displacement ellipsoids drawn at the 30% probability level.

**Fig. 2.**
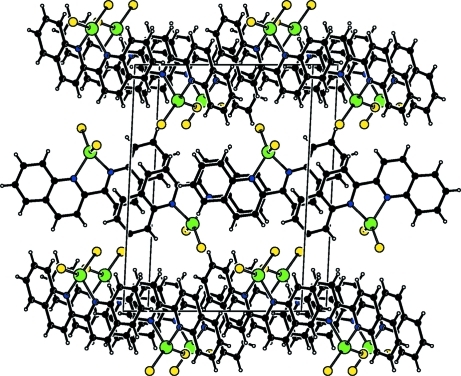
Unit-cell packing diagram for (I).

## supplementary crystallographic information

### Comment

Recently, we reported the synthes and crystal structure of iron (III) hetero-ligand complexes such as [Fe(bipy)Cl_4_][bipy.H], (II), [Fe(5,5'-dmbpy)_2_Cl_2_][FeCl_4_], (III), (Amani, Safari & Khavasi 2007), [Fe(phen)Cl_3_(CH_3_OH)].CH_3_OH, (IV), (Khavasi *et al.*,  2007), [Fe(bipy)Cl_3_(DMSO)], (V) and [Fe(phen)Cl_3_(DMSO)], (VI), (Amani, Safari, khavasi & Mirzaei, 2007), [Fe(phen)Cl_4_][phen.H], (VII), (Khavasi *et al.*,  2008), [Fe(4,4'-dmbpy)Cl_4_][4,4'-dmbpy.H], (VIII) and [Fe(4,4'-dmbpy)Cl_3_(DMSO)], (IX), (Amani *et al.*, 2009) [where bipy is 2,2'-bipyridine, 5,5'-dmbpy is 5,5'-dimethyl-2,2'-bipyridine, phen is 1,10-phenanthroline, DMSO is dimethyl sulfoxide and 4,4'-dmbpy is 4,4'-dimethyl-2,2'-bipyridine].

There are several Fe^II^ complexes, with formula, [FeCl_2_(N—N)], such as [FeCl_2_(6,6'-dmbpy)], (*X*), (Chan & Baird 2004), [FeCl_2_(BDP)], (XI), (Handley *et al.*, 2001) and [FeCl_2_(DEI)], (XII), (Gibson *et al.*, 2002) [where 6,6'-dmbpy is 6, 6'-dimethyl-2, 2'-bipyridine, BDP is 1,3-bis(dimethylamino) propane and DEI is *N*,*N*'-dicyclohexyl-1,2-ethanedi-imine] have been synthesized and characterized by single-crystal X-ray diffraction methods. We report herein the synthesis and crystal structure of the title compound (I).

In the molecule of the title compound, (I), (Fig. 1), the Fe^II^ atom is four-coordinated in distorted tetrahedral configurations by two N atoms from one 2, 2'-biquinoline and two terminal Cl atoms. The Fe—Cl and Fe—N bond lengths and angles (Table 1) are within normal range (*X*). In this complex, Fe—N average distance is 2.1029 (15)Å and the Fe—Cl average bond distance is 2.2303 (6) Å. The Fe—N average bond distances in high-spin Fe^II^ and Fe^III^ phenanthroline and bipyridine complexes are around 2.2 Å. However, low-spin Fe^II^ and Fe^III^ complexes, the Fe—N distances less than 2 Å were reported (Figgis *et al.*, 1983; Kulkarni *et al.*, 1998). Therefore, in the molecule of the title compound, the Fe—N bond distance is unambiguously high-spin Fe^II^. It seems substitution in the 6 position of bipyridine is crucial to stabilize Fe^II^ high-spin *versus* (Fe^II^) especially low-spin. Also, biquinoline result in auto reduce of Fe^III^ to Fe^II^.

The π-π contacts between the pyridine rings, *Cg*2···*Cg*2^i^, *Cg*2···*Cg*4^i^, *Cg*3···*Cg*3^ii^, *Cg*3···*Cg*5^i^, *Cg*4···*Cg*4^ii^ and *Cg*5···*Cg*3^ii^ [symmetry cods: (i) 1-*X*, 1-Y, 1-*Z*, (ii) 1-*X*,-Y,1-*Z*, where *Cg*2, *Cg*3, *Cg*4 and *Cg*5 are centroids of the rings (N1/C1/C6—C9), (N2/C10—C13/C18), (C1—C6) and (C13—C18), respectively] further stabilize the structure, with centroid-centroid distance of 3.7611 (3), 3.7603 (4), 3.5292 (4), 3.5336 (5) and 3.6656 (4) Å, respectively. It seems this π-π stacking is effective in the stabilization of the crystal structure (Fig. 2).

### Experimental

A solution of 2,2'-biquinoline (0.20 g, 0.78 mmol) in methanol (6 ml) and chloroform (2 ml) was added to a solution of FeCl_3_.6H_2_O (0.07 g, 0.26 mmol) in methanol (6 ml) and chloroform (2 ml) and the resulting yellow solution was stirred for 15 min at room temperature. This solution was left to evaporate slowly at room temperature. After two weeks, red blocks of (I) were isolated (yield 0.07 g, 70.3%).

### Refinement

All H atoms were positioned geometrically (C—H = 0.93Å) and refined as riding with *U*_iso_(H)=1.2*U*_eq_.

### Crystal data

**Table:**

[FeCl_2_(C_18_H_12_N_2_)]	*F*(000) = 776
*M**_r_* = 383.05	*D*_x_ = 1.575 Mg m^−^^3^
Monoclinic, *P*2_1_/*n*	Mo *K*α radiation, λ = 0.71073 Å
Hall symbol: -P 2yn	Cell parameters from 1323 reflections
*a* = 7.9777 (6) Å	θ = 2.1–29.3°
*b* = 12.2268 (11) Å	µ = 1.26 mm^−^^1^
*c* = 16.9904 (12) Å	*T* = 298 K
β = 102.899 (6)°	Block, red
*V* = 1615.5 (2) Å^3^	0.45 × 0.43 × 0.31 mm
*Z* = 4	

### Data collection

**Table:**

Stoe IPDS II diffractometer	4323 independent reflections
Radiation source: fine-focus sealed tube	3739 reflections with *I* > 2σ(*I*)
graphite	*R*_int_ = 0.024
Detector resolution: 0.15 mm pixels mm^-1^	θ_max_ = 29.3°, θ_min_ = 2.1°
rotation method scans	*h* = −10→10
Absorption correction: numerical (*X-SHAPE*; Stoe & Cie, 2005)	*k* = −16→16
*T*_min_ = 0.577, *T*_max_ = 0.681	*l* = −23→21
13058 measured reflections	

### Refinement

**Table:**

Refinement on *F*^2^	Primary atom site location: structure-invariant direct methods
Least-squares matrix: full	Secondary atom site location: difference Fourier map
*R*[*F*^2^ > 2σ(*F*^2^)] = 0.037	Hydrogen site location: inferred from neighbouring sites
*wR*(*F*^2^) = 0.093	H-atom parameters constrained
*S* = 1.06	*w* = 1/[σ^2^(*F*_o_^2^) + (0.0407*P*)^2^ + 0.7232*P*] where *P* = (*F*_o_^2^ + 2*F*_c_^2^)/3
4323 reflections	(Δ/σ)_max_ = 0.007
208 parameters	Δρ_max_ = 0.52 e Å^−^^3^
0 restraints	Δρ_min_ = −0.44 e Å^−^^3^

### Special details

**Table:**

Geometry. All e.s.d.'s (except the e.s.d. in the dihedral angle between two l.s. planes) are estimated using the full covariance matrix. The cell e.s.d.'s are taken into account individually in the estimation of e.s.d.'s in distances, angles and torsion angles; correlations between e.s.d.'s in cell parameters are only used when they are defined by crystal symmetry. An approximate (isotropic) treatment of cell e.s.d.'s is used for estimating e.s.d.'s involving l.s. planes.
Refinement. Refinement of *F*^2^ against ALL reflections. The weighted *R*-factor *wR* and goodness of fit *S* are based on *F*^2^, conventional *R*-factors *R* are based on *F*, with *F* set to zero for negative *F*^2^. The threshold expression of *F*^2^ > σ(*F*^2^) is used only for calculating *R*-factors(gt) *etc*. and is not relevant to the choice of reflections for refinement. *R*-factors based on *F*^2^ are statistically about twice as large as those based on *F*, and *R*- factors based on ALL data will be even larger.

### Fractional atomic coordinates and isotropic or equivalent isotropic displacement parameters (Å^2^)

**Table:**

	*x*	*y*	*z*	*U*_iso_*/*U*_eq_	
C1	0.2274 (2)	0.95885 (14)	0.02892 (11)	0.0367 (3)	
C2	0.3108 (3)	1.01753 (16)	0.09821 (13)	0.0462 (4)	
H2	0.3097	0.9909	0.1494	0.055*	
C3	0.3929 (3)	1.11314 (18)	0.09004 (15)	0.0546 (5)	
H3	0.4477	1.1515	0.1359	0.066*	
C4	0.3959 (3)	1.15461 (17)	0.01308 (17)	0.0571 (6)	
H4	0.4536	1.2196	0.0086	0.069*	
C5	0.3157 (3)	1.10094 (17)	−0.05409 (15)	0.0520 (5)	
H5	0.3174	1.1297	−0.1046	0.062*	
C6	0.2289 (2)	1.00112 (15)	−0.04852 (12)	0.0417 (4)	
C7	0.1447 (3)	0.94060 (17)	−0.11614 (12)	0.0475 (4)	
H7	0.1447	0.9656	−0.1678	0.057*	
C8	0.0630 (3)	0.84538 (16)	−0.10617 (11)	0.0438 (4)	
H8	0.0069	0.8052	−0.1508	0.053*	
C9	0.0647 (2)	0.80865 (14)	−0.02736 (10)	0.0352 (3)	
C10	−0.0289 (2)	0.70846 (13)	−0.01190 (10)	0.0352 (3)	
C11	−0.1412 (3)	0.65279 (15)	−0.07475 (11)	0.0426 (4)	
H11	−0.1539	0.6755	−0.1280	0.051*	
C12	−0.2310 (2)	0.56528 (16)	−0.05670 (12)	0.0459 (4)	
H12	−0.3060	0.5280	−0.0977	0.055*	
C13	−0.2108 (2)	0.53109 (15)	0.02390 (12)	0.0423 (4)	
C14	−0.2993 (3)	0.44022 (17)	0.04699 (16)	0.0550 (5)	
H14	−0.3764	0.4011	0.0080	0.066*	
C15	−0.2720 (3)	0.4098 (2)	0.12585 (17)	0.0637 (6)	
H15	−0.3316	0.3506	0.1404	0.076*	
C16	−0.1546 (4)	0.4673 (2)	0.18538 (16)	0.0652 (6)	
H16	−0.1362	0.4450	0.2390	0.078*	
C17	−0.0666 (3)	0.55570 (18)	0.16561 (13)	0.0544 (5)	
H17	0.0105	0.5933	0.2056	0.065*	
C18	−0.0937 (2)	0.58938 (15)	0.08446 (11)	0.0398 (4)	
N1	0.14684 (19)	0.86245 (11)	0.03785 (8)	0.0349 (3)	
N2	−0.00420 (19)	0.67763 (12)	0.06478 (8)	0.0361 (3)	
Fe1	0.17508 (4)	0.77276 (2)	0.145770 (14)	0.04109 (9)	
Cl1	0.43989 (8)	0.70190 (6)	0.17323 (4)	0.06732 (17)	
Cl2	0.05960 (9)	0.84372 (5)	0.24216 (3)	0.06434 (17)	

### Atomic displacement parameters (Å^2^)

**Table:**

	*U*^11^	*U*^22^	*U*^33^	*U*^12^	*U*^13^	*U*^23^
C1	0.0346 (8)	0.0354 (8)	0.0411 (9)	0.0060 (7)	0.0107 (7)	0.0011 (6)
C2	0.0462 (10)	0.0451 (10)	0.0478 (10)	−0.0023 (8)	0.0114 (8)	−0.0066 (8)
C3	0.0477 (11)	0.0463 (10)	0.0710 (14)	−0.0045 (9)	0.0156 (10)	−0.0112 (10)
C4	0.0507 (12)	0.0392 (10)	0.0878 (17)	−0.0023 (9)	0.0289 (12)	0.0013 (10)
C5	0.0519 (11)	0.0429 (10)	0.0679 (13)	0.0073 (9)	0.0278 (10)	0.0135 (9)
C6	0.0409 (9)	0.0401 (9)	0.0479 (10)	0.0089 (7)	0.0177 (8)	0.0062 (7)
C7	0.0558 (11)	0.0511 (11)	0.0372 (9)	0.0099 (9)	0.0142 (8)	0.0113 (8)
C8	0.0535 (11)	0.0459 (9)	0.0307 (8)	0.0044 (8)	0.0066 (7)	0.0020 (7)
C9	0.0385 (8)	0.0362 (8)	0.0302 (7)	0.0073 (7)	0.0062 (6)	0.0016 (6)
C10	0.0360 (8)	0.0359 (8)	0.0319 (7)	0.0055 (6)	0.0041 (6)	−0.0021 (6)
C11	0.0446 (10)	0.0443 (9)	0.0341 (8)	0.0052 (8)	−0.0018 (7)	−0.0032 (7)
C12	0.0391 (9)	0.0438 (9)	0.0492 (10)	0.0022 (8)	−0.0017 (8)	−0.0082 (8)
C13	0.0357 (9)	0.0388 (9)	0.0537 (11)	0.0023 (7)	0.0127 (8)	−0.0036 (8)
C14	0.0449 (11)	0.0452 (10)	0.0780 (15)	−0.0050 (9)	0.0205 (10)	−0.0055 (10)
C15	0.0647 (14)	0.0508 (12)	0.0867 (18)	−0.0081 (11)	0.0409 (13)	0.0016 (11)
C16	0.0862 (18)	0.0610 (13)	0.0585 (13)	−0.0056 (13)	0.0374 (13)	0.0073 (11)
C17	0.0693 (14)	0.0544 (12)	0.0433 (10)	−0.0083 (10)	0.0204 (10)	0.0008 (9)
C18	0.0411 (9)	0.0383 (8)	0.0422 (9)	0.0008 (7)	0.0138 (7)	−0.0006 (7)
N1	0.0383 (7)	0.0352 (7)	0.0310 (6)	0.0033 (6)	0.0074 (5)	0.0004 (5)
N2	0.0398 (7)	0.0372 (7)	0.0308 (6)	0.0008 (6)	0.0070 (5)	−0.0009 (5)
Fe1	0.04860 (17)	0.04469 (15)	0.02751 (13)	−0.00218 (11)	0.00321 (10)	0.00021 (10)
Cl1	0.0545 (3)	0.0818 (4)	0.0594 (3)	0.0111 (3)	−0.0006 (2)	0.0081 (3)
Cl2	0.0820 (4)	0.0767 (4)	0.0333 (2)	0.0170 (3)	0.0107 (2)	−0.0042 (2)

### Geometric parameters (Å, °)

**Table:**

C1—N1	1.367 (2)	C11—C12	1.360 (3)
C1—C2	1.412 (3)	C11—H11	0.9300
C1—C6	1.416 (3)	C12—C13	1.406 (3)
C2—C3	1.362 (3)	C12—H12	0.9300
C2—H2	0.9300	C13—C18	1.418 (3)
C3—C4	1.407 (4)	C13—C14	1.418 (3)
C3—H3	0.9300	C14—C15	1.360 (4)
C4—C5	1.348 (3)	C14—H14	0.9300
C4—H4	0.9300	C15—C16	1.404 (4)
C5—C6	1.417 (3)	C15—H15	0.9300
C5—H5	0.9300	C16—C17	1.371 (3)
C6—C7	1.405 (3)	C16—H16	0.9300
C7—C8	1.363 (3)	C17—C18	1.409 (3)
C7—H7	0.9300	C17—H17	0.9300
C8—C9	1.409 (2)	C18—N2	1.376 (2)
C8—H8	0.9300	Fe1—N1	2.1051 (14)
C9—N1	1.329 (2)	Fe1—N2	2.1008 (15)
C9—C10	1.488 (2)	Fe1—Cl2	2.2265 (6)
C10—N2	1.328 (2)	Fe1—Cl1	2.2341 (7)
C10—C11	1.407 (2)		
			
N1—C1—C2	119.43 (16)	C11—C12—C13	120.10 (17)
N1—C1—C6	121.30 (16)	C11—C12—H12	119.9
C2—C1—C6	119.27 (17)	C13—C12—H12	119.9
C3—C2—C1	119.9 (2)	C12—C13—C18	118.12 (17)
C3—C2—H2	120.0	C12—C13—C14	123.11 (19)
C1—C2—H2	120.0	C18—C13—C14	118.76 (19)
C2—C3—C4	120.8 (2)	C15—C14—C13	120.4 (2)
C2—C3—H3	119.6	C15—C14—H14	119.8
C4—C3—H3	119.6	C13—C14—H14	119.8
C5—C4—C3	120.5 (2)	C14—C15—C16	120.5 (2)
C5—C4—H4	119.7	C14—C15—H15	119.8
C3—C4—H4	119.7	C16—C15—H15	119.8
C4—C5—C6	120.6 (2)	C17—C16—C15	120.9 (2)
C4—C5—H5	119.7	C17—C16—H16	119.5
C6—C5—H5	119.7	C15—C16—H16	119.5
C7—C6—C1	117.76 (17)	C16—C17—C18	119.7 (2)
C7—C6—C5	123.42 (19)	C16—C17—H17	120.2
C1—C6—C5	118.83 (19)	C18—C17—H17	120.2
C8—C7—C6	120.19 (17)	N2—C18—C17	119.52 (17)
C8—C7—H7	119.9	N2—C18—C13	120.76 (16)
C6—C7—H7	119.9	C17—C18—C13	119.71 (18)
C7—C8—C9	119.12 (18)	C9—N1—C1	119.39 (15)
C7—C8—H8	120.4	C9—N1—Fe1	113.92 (11)
C9—C8—H8	120.4	C1—N1—Fe1	125.75 (11)
N1—C9—C8	122.20 (17)	C10—N2—C18	119.32 (15)
N1—C9—C10	115.71 (14)	C10—N2—Fe1	114.56 (12)
C8—C9—C10	122.08 (16)	C18—N2—Fe1	126.12 (12)
N2—C10—C11	122.49 (17)	N2—Fe1—N1	78.06 (6)
N2—C10—C9	115.87 (14)	N2—Fe1—Cl2	111.38 (5)
C11—C10—C9	121.60 (15)	N1—Fe1—Cl2	117.15 (4)
C12—C11—C10	119.19 (17)	N2—Fe1—Cl1	113.32 (5)
C12—C11—H11	120.4	N1—Fe1—Cl1	107.24 (5)
C10—C11—H11	120.4	Cl2—Fe1—Cl1	121.65 (3)
			
N1—C1—C2—C3	179.35 (18)	C12—C13—C18—N2	0.7 (3)
C6—C1—C2—C3	−0.6 (3)	C14—C13—C18—N2	179.59 (17)
C1—C2—C3—C4	0.0 (3)	C12—C13—C18—C17	−178.33 (19)
C2—C3—C4—C5	0.7 (3)	C14—C13—C18—C17	0.5 (3)
C3—C4—C5—C6	−0.8 (3)	C8—C9—N1—C1	2.5 (3)
N1—C1—C6—C7	0.0 (3)	C10—C9—N1—C1	−176.11 (14)
C2—C1—C6—C7	179.98 (17)	C8—C9—N1—Fe1	−167.08 (14)
N1—C1—C6—C5	−179.45 (16)	C10—C9—N1—Fe1	14.32 (19)
C2—C1—C6—C5	0.5 (3)	C2—C1—N1—C9	178.34 (17)
C4—C5—C6—C7	−179.2 (2)	C6—C1—N1—C9	−1.7 (2)
C4—C5—C6—C1	0.2 (3)	C2—C1—N1—Fe1	−13.4 (2)
C1—C6—C7—C8	0.9 (3)	C6—C1—N1—Fe1	166.54 (13)
C5—C6—C7—C8	−179.67 (19)	C11—C10—N2—C18	−1.3 (3)
C6—C7—C8—C9	−0.2 (3)	C9—C10—N2—C18	176.38 (15)
C7—C8—C9—N1	−1.6 (3)	C11—C10—N2—Fe1	178.50 (13)
C7—C8—C9—C10	176.93 (17)	C9—C10—N2—Fe1	−3.78 (19)
N1—C9—C10—N2	−7.2 (2)	C17—C18—N2—C10	179.36 (18)
C8—C9—C10—N2	174.23 (17)	C13—C18—N2—C10	0.3 (3)
N1—C9—C10—C11	170.57 (16)	C17—C18—N2—Fe1	−0.4 (3)
C8—C9—C10—C11	−8.0 (3)	C13—C18—N2—Fe1	−179.52 (13)
N2—C10—C11—C12	1.3 (3)	C10—N2—Fe1—N1	8.54 (12)
C9—C10—C11—C12	−176.28 (17)	C18—N2—Fe1—N1	−171.64 (15)
C10—C11—C12—C13	−0.2 (3)	C10—N2—Fe1—Cl2	123.25 (12)
C11—C12—C13—C18	−0.8 (3)	C18—N2—Fe1—Cl2	−56.93 (15)
C11—C12—C13—C14	−179.55 (19)	C10—N2—Fe1—Cl1	−95.27 (12)
C12—C13—C14—C15	178.8 (2)	C18—N2—Fe1—Cl1	84.55 (14)
C18—C13—C14—C15	0.0 (3)	C9—N1—Fe1—N2	−12.47 (12)
C13—C14—C15—C16	−0.7 (4)	C1—N1—Fe1—N2	178.74 (14)
C14—C15—C16—C17	0.8 (4)	C9—N1—Fe1—Cl2	−120.54 (11)
C15—C16—C17—C18	−0.3 (4)	C1—N1—Fe1—Cl2	70.66 (14)
C16—C17—C18—N2	−179.5 (2)	C9—N1—Fe1—Cl1	98.51 (12)
C16—C17—C18—C13	−0.4 (3)	C1—N1—Fe1—Cl1	−70.28 (14)
